# Primary Breast Mucormycosis, A Case Report

**Published:** 2011-03-01

**Authors:** S R Baezzat, A Fazelzadeh, S Tahmasebi, P V Kumar

**Affiliations:** 1 Department of General Surgery, Shahid Faghihi Hospital, Shiraz University of Medical Sciences, Shiraz, Iran; 2 Department of Pathology, Shiraz University of Medical Sciences, Shiraz, Iran

**Keywords:** Breast, Mucormycosis, Primary, Iran

## Abstract

Mucormycosis is a rare fungal infection caused by organisms of the order Mucorales often occurring in diabetics or immunologically compromised patients. To date, only one case of primary mucormycosis of breast has been reported in the English literature. We describe a further case of mucormycosis that was localized to the breast in a patient with no underlying disease and was successfully treated with a simple mastectomy and intravenous antifungal therapy.

## Introduction

Mucormycosis is a rare fungal infection caused by organisms of the order Mucorales often occurring in diabetics or immunologically compromised patients but the individuals without an obvious underlying predisposing factor can rarely be involved with this disease.[1]

Once infection has occurred, the fungus invade the vasculature and leads to thrombosis and tissue ischemia and finally necrosis with a high mortality rate.[2][3] With aggressive surgical debridement, control of comorbidities such as hyperglycemia and immunosuppression, and the use of modern antifungal agents, survival rates for invasive mucormycosis are 36% to 50%.[4]

To date, only one case of primary mucormycosis of breast has been reported in the English literature.[5] We describe a further case of mucormycosis that was localized to the breast in a patient with no underlying disease and was successfully treated with a simple mastectomy and intravenous antifungal therapy.

## Case Report

**Fig. 1:**
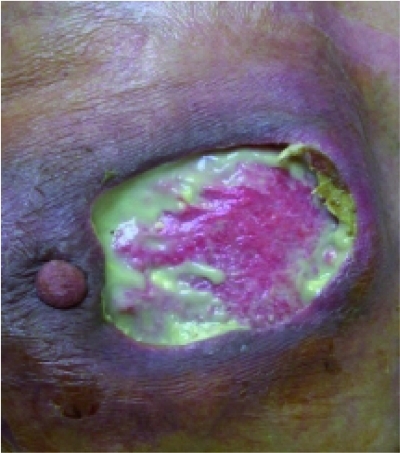
Incision was taken over the breast to drain underlying pus collections and debridement of necrotic tissue of breast was done.

A 75 year old female presented gradual onset of pain, redness and swelling of the left breast with 7 days duration without any history of trauma, diabetes mellitus or other chronic diseases. She did not take any medication. On examination, she was febrile and had pulse rate of 90 beats/minute and blood pressure of 110/80 mmHg. Her left breast was swollen, red, tender and warm with a necrotic lesion of 3x4 cm. Investigations revealed a hemoglobin concentration of 12 gm% with a white cell count of 18,500/cu.mm. Renal and liver function tests were within normal limits as was the chest radiograph. A diagnosis of severe mastitis with probable abscess was made. An incision was taken over the breast to drain the underlying pus collections if any but there were no localized abscess (Figure 1).

**Fig. 2:**
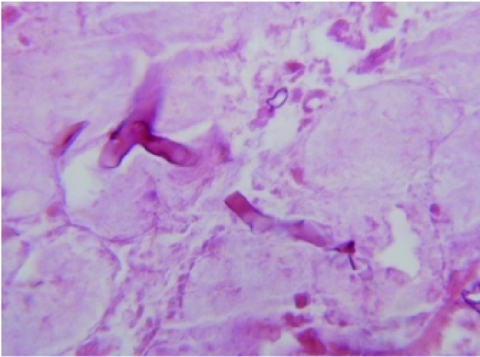
Histopathological findings. Microscopic view of the biopsy specimen shows several short folded hyphae with non-septate broad and right angle buddings which are characteristic for mucormycosis. PAS was negative (H and E, 1200X)

However, within 24 hours the underlying breast tissue showed necrosis with black eschar formation. An incisional biopsy revealed breast parenchymal necrosis, polymorphonuclear infiltration and several broad aseptate hyaline fungal hyphae. A simple mastectomy was done. Histopathological examination showed extensive necrosis of the breast parenchyma with polymorphonuclear infiltration, hemorrhage and thrombosed blood vessels and many broad aseptate hyaline fungal hyphae branching at 90o suggestive of mucormycosis (Figure 2).

Intravenous amphotericin-B (1 mg/kg/day in the form of infusion in 5% dextrose) was administered for 10 days followed by one month fluconazole treatment. The patient showed complete remission after one week. We grafted the wound with split thickness skin graft after one month. She was asymptomatic during her 3 months follow up.

## Discussion

Mucormycosis is caused by various members of the phycomycetes, such as rhizopus, mucor, and absidia.[6]They are usually found in soil, spoiled foods, bread, and dust, so most individuals are exposed to these fungi on a daily basis but most often diabetics or patients with immunosuppressed conditions like chronic steroid use, metabolic acidosis, organ transplantation, leukemia/lymphoma, treatment with deferoxamine, and AIDS are susceptible to this infection.[1]

In our patient, spores were likely to have gained entry to breast through unknown trauma and multiplied and invaded the breast tissue. The predisposing factor making our patient susceptible to this infection was not clear. Old age is the only factor which may result to a immunocompromised situation. Althoughn there are some reports in the literature that patients without underlying disease may develop mucormycosis too.[1] Mucormycosis often manifests as a rhinoorbitocerebral, pulmonary, gastrointestinal, cutaneous, or disseminated disease.[1] There are cases of cutaneous mucormycosis after minor trauma but these patients had diabetes as an underlying disease.[7][8]

Invasive surgical debridement with systemic amphotericin B led to a favorable response in our patient. To our knowledge, only one other report of primary breast mucormycosis has been reported.[5] In that report, they used simple mastectomy followed by amphotricin B therapy, as we did, and their result was successful.
